# A novel oxygen carrier “YQ23” suppresses the liver tumor metastasis by decreasing circulating endothelial progenitor cells and regulatory T cells

**DOI:** 10.1186/1471-2407-14-293

**Published:** 2014-04-27

**Authors:** Chang Xian Li, Bing L Wong, Chang Chun Ling, Yuen Yuen Ma, Yan Shao, Wei Geng, Xiang Qi, Sze Hang Lau, Sui Yi Kwok, Na Wei, Fei Chuen Tzang, Kevin TP Ng, Xiao Bing Liu, Chung Mau Lo, Kwan Man

**Affiliations:** 1 Department of Surgery and Centre for Cancer Research, LKS Faculty of Medicine, The University of Hong Kong, Hong Kong, China; 2 New B Innovation Limited, Hong Kong, China

**Keywords:** YQ23, HCC, Tumor metastasis, Hepatic I/R injury, EPCs, Tregs

## Abstract

**Background:**

Surgical therapies are the first-line treatments for hepatocellular carcinoma (HCC) patients. However, the high incidence of tumor metastasis after liver surgery remains a severe problem. We aim to investigate the roles and the underlying mechanism of YQ23, stabilized non-polymeric diaspirin cross-linked tetrameric hemoglobin, in liver tumor metastasis after major hepatectomy and partial hepatic ischemia reperfusion (I/R) injury.

**Methods:**

An orthotopic liver tumor model in Buffalo rat was established using the hepatocellular carcinoma cell line McA-RH7777. Major hepatectomy for tumor-bearing lobe and partial hepatic I/R injury were performed at two weeks after orthotopic liver tumor implantation. YQ23 (0.2 g/kg) was administered at 1 hour before ischemia and immediately after reperfusion. Blood samples were collected at day 0, 1, 7, 14, 21 and 28 for detection of circulating endothelial progenitor cells (EPCs) and regulatory T cells (Tregs).

**Results:**

Our results showed that YQ23 treatment effectively inhibited intrahepatic and lung metastases together with less tumor angiogenesis at 4 weeks after major hepatectomy and partial hepatic I/R injury. The levels of circulating EPCs and Tregs were significantly decreased in YQ23 treatment group. Furthermore, YQ23 treatment also increased liver tissue oxygenation during hepatic I/R injury. Up-regulation of HO1 and down-regulation of CXCR3, TNF-α and IL6 were detected after YQ23 treatment.

**Conclusions:**

YQ23 treatment suppressed liver tumor metastasis after major hepatectomy and partial hepatic I/R injury in a rat liver tumor model through increasing liver oxygen and reducing the populations of circulating EPCs and Tregs.

## Background

Hepatocellular carcinoma (HCC) is one of the most common malignancies in the world, claiming 600,000 victims each year [1]. Although remarkable advances in diagnostic and non-surgical therapeutic techniques have been made in the recent years, surgical therapies such as liver resection and liver transplantation are still the first-line treatments for HCC patients. However, the high incidence of tumor metastasis after liver surgery remains a major problem [2,3]. Therefore, it is very important to develop novel therapies to reduce liver tumor metastasis after liver surgery.

Surgical stress injury such as liver ischemia and hypoxia are inevitable consequence during liver surgery. Accumulating evidence suggested that tissue ischemia and hypoxia can rapidly increase the number of circulating EPCs and Tregs [4-6]. These events are also associated with elevated levels of hypoxia inducible factor-1*α* (HIF-1*α*) responsive chemokines and inflammatory cytokines/chemokines that stimulate the release and recruitment of EPCs and Trges from the bone marrow [4,6-10]. EPCs play important roles in tumor vasculogenesis and tumor growth at early phase by providing structural support to nascent vessels and the release of pro-angiogenic cytokines [11,12]. Furthermore, EPCs have major roles in control angiogenic switch of metastasis transition from micrometastases to macrometastases [13]. CD4^+^CD25^+^Foxp3^+^ Tregs play a critical role in the control of antitumor immune responses [14,15]. It has been found that increased numbers of Tregs are detected in peripheral blood of cancer patients and accumulate in tumor regions [16-18].

YQ product is the stabilized non-polymeric cross-linked tetrameric hemoglobin (65 kDa) with undetectable/low level of dimeric hemoglobin (32 kDa), phospholipid, DNA impurities and protein impurities. Recent research showed that the similar product OC89 can effectively increase the ability to carry oxygen and enhance cisplatin-based TACE [19]. In this study, we aimed to investigate whether the treatment of YQ23 can suppress liver tumor metastasis after major hepatectomy and partial hepatic I/R injury by increasing liver oxygen and then reducing circulating EPCs and Tregs levels in an orthotopic rat liver tumor model. The significance of this study will hopefully provide a novel means to suppress liver tumor metastasis after liver surgery for HCC patients.

## Methods

### Animal model

Buffalo rats (Male, 8-10weeks, 350-400 g) were obtained from lab animal unit, The University of Hong Kong. Before operation, rats were anesthesia with pentobarbitone (40 mg/kg, ip) and after operation buprenorphine (0.05-0.1 mg/kg/12 hours) were given for attenuating the analgesics. Rats were housed in a standard animal laboratory with free activity and access to water and food. They were kept under constant environment conditions with a 12-hour light–dark cycle. All operations were performed under clean conditions. The study had been licensed according to Animal Ordinance Chapter 340 by the Department of Health, Hong Kong Special Administrative Region (ref.: (12–222) in DH/HA&P/8/2/3 Pt. 39). Hepatocellular carcinoma cell line McA-RH7777 (Purchased from the American Type Culture Collection, Number CRL1601, ATCC, Manassas, VA, USA) were used to establish the orthotopic liver cancer model in Buffalo rat [20]. Two weeks after orthotopic liver tumor implantation, The branch of hepatic artery and portal vein to right and triangle lobes are clamped for 30 minutes (ischemia duration) following by reperfusion (release of the clamp). Major hepatectomy of tumor-bearing lobe (left lobe) is performed during the ischemia duration.

### Treatment regimen and sample collection

YQ23 products were obtained from New B Innovation Limited. YQ23 product is the stabilized non-polymeric cross-linked tetrameric hemoglobin (65 kDa) with undetectable/low level of dimeric hemoglobin (32 kDa), phospholipid, DNA impurities and protein impurities. The concentration of YQ product is 10 g/dL and its pH range is 7.2-7.8. The osmolality and viscosity (at 37°C) are >250 mOsm/kg and 0.9 centipoise respectively. The p50 value is ~ 40 mmHg. The information for YQ product is shown in patent no. US7,932,356 B1, US 8,048,856 B1 and PCT/US12/46130. YQ23 (0.2 g/kg, treatment group) or the same volume of ringer’s acetate buffer (control group) were administrated intravenously at 1 hour before ischemia. An additional 0.2 g/kg YQ23 or the same volume of ringer’s acetate buffer was injected through hepatic portal vein immediately after reperfusion. Blood samples were taken at days 0, 1, 7, 14, 21 and 28 from tail vein for detecting the populations of circulating EPCs and Tregs. And then Buffalo rats were sacrificed at 4 weeks after major hepatectomy and partial hepatic I/R injury to monitor liver tumor metastasis. Liver and lung were sampled for further investigation.

### Hematoxylin and Eosin (H & E) and Immunohistochemical (IHC) Staining

The histological changes were detected by H& E staining and the expression of CD34 (Santa Cruz Biotechnology) were detected by immunohistochemical staining. The details of H & E and IHC staining were described in our previous paper [20]. In brief, after rehydrated in water, the paraffin sections placed in citric buffer (pH 6.0) and treated in a microwave. Afterwards, the sections underwent blocking with 10% FBS and then primary antibodies were applied (incubated at 4°C overnight). Then the sections underwent blocking with 3% peroxidase for 30 min and secondary antibodies from Dako EnVision System (DakoCytomation) were applied. Signals were developed with 3,3’-diaminobenzidine substrate solution (DakoCytomation).

### Determination of microvessel density and tumor load analyses

Microvessel density (MVD) of intrahepatic metastatic tumor sections was evaluated [21]. The mean number of tumor nodules in the lungs and liver, as well as tumor volume of intrahepatic metastatic nodules (L × W^2^/2) was calculated and expressed as average [22].

### Detection of circulating EPCs and Tregs by flow cytometry

The number of circulating EPCs and Tregs were detected by flow cytometry. The details of flow cytometry were described in our previous paper [20]. For analysis of EPC cell surface molecules, cells were stained with the following antibodies: unconjugated rabbit anti-CD133 (Abcam, Cambridge, UK), PE-conjugated anti-CD34 (Santa Cruz Biotech, Santa Cruz, CA), VEGFR2^+^ (BD Pharmingen, San Diego, CA) and goat anti-rabbit FITC secondary antibody (Abcam, Cambridge, UK). For analysis of Treg cells, cells were stained with PE-Cy5-conjugated anti CD4 (eBioscience, San Diego, CA) and PE-conjugated anti CD25 (BD Pharmingen, San Diego, CA), and then permeabilized with fixation/permeabiliation working solution and incubated with FITC-conjugated anti-Foxp3 (eBioscience, San Diego, CA).

### Determination of liver oxygenation

Liver tissue oxygenation (Liver pO_2_) was directly monitored by the OxyLab® *in vivo* monitoring system (Oxford Optronix, UK) during the ischemia and reperfusion procedures in another group of Buffalo rats. Briefly, a large-area-surface (LAS) oxygen sensor (Oxford Optronix, UK) was placed between the right hepatic lobe and triangle lobe of the rat livers. The branch of hepatic artery and portal vein to right and triangle lobes were clamped for 30 minutes following by reperfusion. YQ23 (0.2 g/kg, treatment group) or ringer’s acetate buffer (control group) were administered intravenously at 1 hour before ischemia. An additional 0.2 g/kg YQ23 or ringer’s acetate buffer was injected through hepatic portal vein immediately after reperfusion. Liver oxygen tension was continuously measured during hepatic ischemia reperfusion injury: 1) baseline; 2) after infusion of YQ23 or ringer’s acetate buffer; 3) during ischemia; and 4) after onset of reperfusion.

### Assessment of hepatic gene expression profiles

In order to investigate the effect of YQ23 on expressions of cytokines/chemokines, a new group of Buffalo rats were included for major hepatecotmy and partial hepatic I/R injury. YQ23 or ringer’s acetate buffer were administered at 1 hour before ischemia and immediately after reperfusion. Liver samples were collected at 6 hours after reperfusion and gene expressions were detected by reverse transcription-polymerase chain reaction (RT-PCR) [20]. Gene expression levels were expressed as the folds relative to the normal liver. The sequences of the primers were listed as follows: CXCR3: Left AGCACAGACACCTTCCTGCT, Right CAGAGACCAGAGCCGAAAAC; TNF-α: Left GTCTGTGCCTCAGCCTCTTC, Right CCCATTTGGGAACTTCTCCT. IL6: Left GCCCTTCAGGAACAGCTATG, Right GTCTCCTCTCCGGACTTGTG; HO1: Left GAAGAAGATTGCGCAGAAGG, Right TTCATGCGAGCACGATAGAG.

### Statistics and data analyses

Continuous variables were expressed as average. T-TEST was applied to delineate the difference between the treatment and control groups. Chi-Square (χ2) test was used to compare incidence of intrahepatic and lung metastasis after major hepatectomy and partial hepatic I/R injury. *p <* 0.05 was considered statistically significant. Calculations were performed by using the SPSS computer software version 16 (SPSS, Chicago, IL).

## Results

### YQ23 suppressed liver tumor metastasis after major hepatectomy and partial hepatic I/R injury

**Figure 1:**
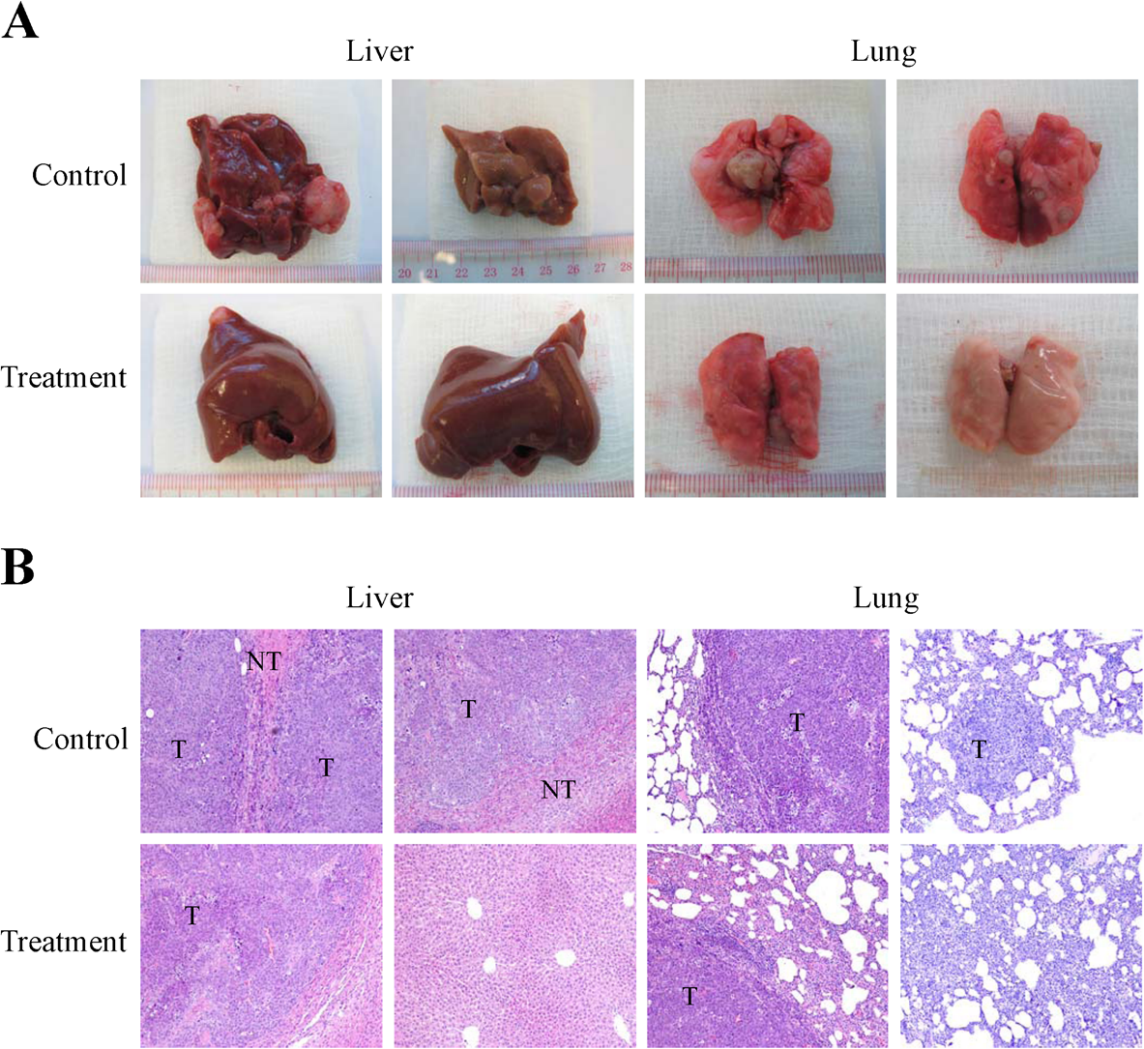
**YQ23 suppressed liver tumor intrahepatic and lung metastasis after major hepatectomy and partial I/R injury.** Buffalo rats were sacrificed at 4 weeks after major hepatectomy and hepatic I/R injury to monitor tumor metastasis. **(A)** Intrahepatic and lung metastasis were detected in specimen at four weeks after major hepatectomy and partial I/R injury. **(B)** Histological features of intrahepatic and lung metastasis tumors at four weeks after hepatectomy and partial I/R injury with and without YQ23 treatment. (T = tumor; NT = Non-tumor).

In order to investigate the effect of YQ23 on the metastasis of liver tumor after liver surgery, we established a rat orthotopic liver tumor model with local and distant metastatic potentials. The results showed that, after YQ23 treatment, the incidence of intrahepatic metastasis reduced from 69.2% (9 of 13) to 36.4% (4 of 11). Similarly, YQ23 treatment also decreased the lung metastasis from 53.8% (7 of 13) to 36.4% (4 of 11) (Table 1). More importantly, the tumor volume of intrahepatic metastatic nodules was significantly decreased in YQ23 treatment group (0.201 cm^3^*vs* 0.059 cm^3^, *p = 0.045*) (Table 1 and Figure 1A). Furthermore, the number of metastatic tumor nodules in liver and lung were also reduced after YQ23 treatment (intrahepatic metastasis: 3.5 *vs* 0.6, *p = 0.15*; lung metastasis: 2.2 *vs* 1.0, *p = 0.4*) (Table 1). Intrahepatic and lung metastasis were further confirmed by histology examination (Figure 1B).

**Table 1:** Comparison of liver tumor metastasis after major hepatecotmy and partial hepatic I/R injury

	**Control group**	**Treatment group**	***p *** **value**
Intrahepatic metastasis			
Metastasis ratio	9/13(69.2%)	4/11(36.4%)	0.107
Tumor size(cm^3^)	0.201 ± 0.058	0.059 ± 0.029	0.045*
Number(tumor nodules)/rat	3.539 ± 1.767	0.636 ± 0.310	0.151
Lung metastasis			
Metastasis ratio	7/13(53.8%)	4/11(36.4%)	0.392
Number(tumor nodules)/rat	2.154 ± 1.143	1.00 ± 0.539	0.399

Note: average ± SEM *p < 0.05.

### YQ23 decreased the MVD in intrahepatic metastatic tumor nodules

**Figure 2:**
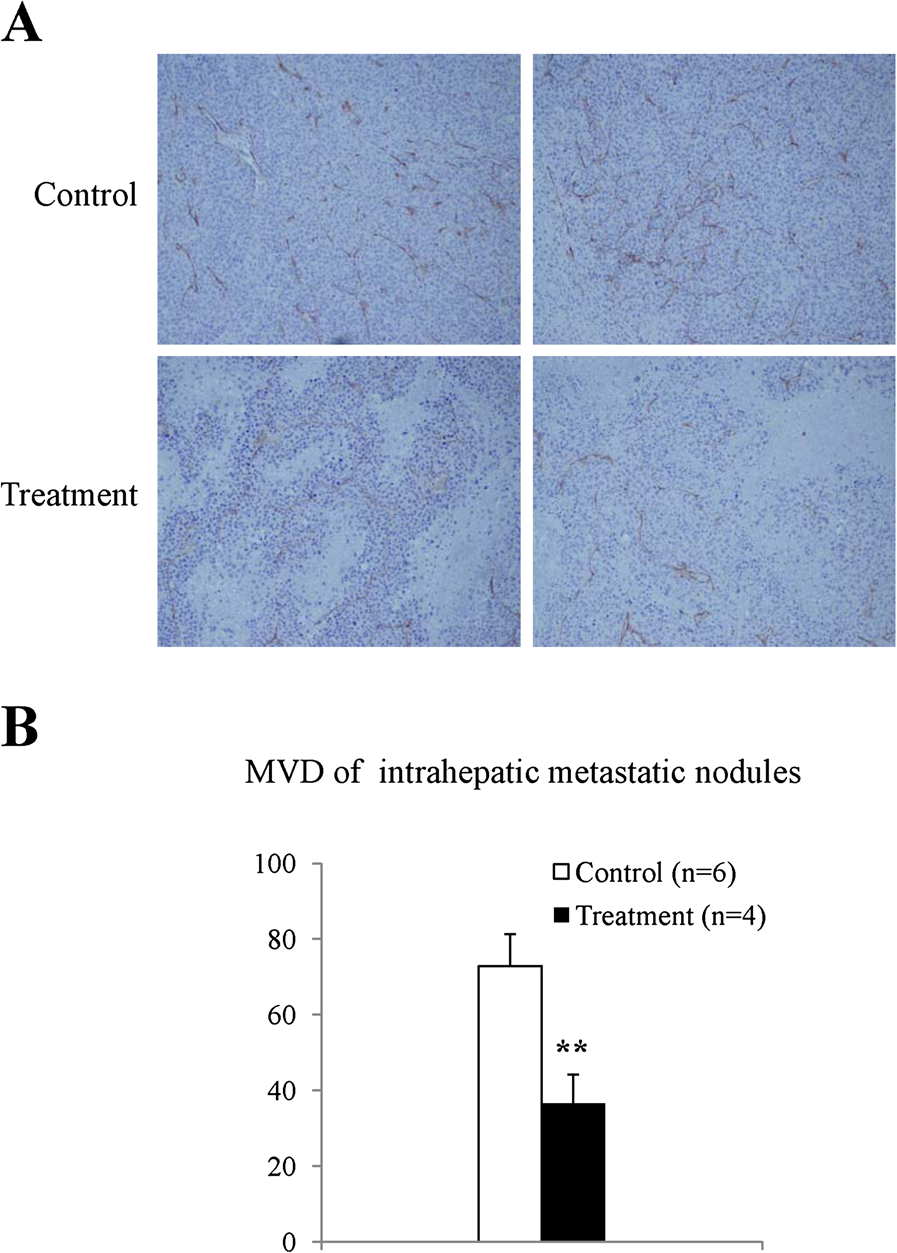
**Comparison of tumor angiogenesis in intrahepatic metastatic tumor.** CD34 positive cells in intrahepatic metastatic tumor nodules were detected by IHC staining. **(A)** The expression of CD34 positive cells in intrahepatic metastatic tumor nodules. **(B)** Comparison of MVD in metastatic tumor nodules between YQ23 group and control group. (**p < 0.01; Treatment group N = 4; Control group N = 6).

At four weeks after major hepatectomy and partial hepatic I/R injury, IHC staining revealed that strong expression of CD34 in intrahepatic metastatic tumor nodules were detected in the control group. On the contrary, only weak CD34 expression was found in the YQ23 treatment group (Figure 2A). Furthermore, YQ23 treatment significantly reduced MVD in intrahepatic metastatic tumor nodules (36.4 *vs* 72.96; *p < 0.01*) (Figure 2B).

### YQ23 significantly reduced circulating EPCs

**Figure 3:**
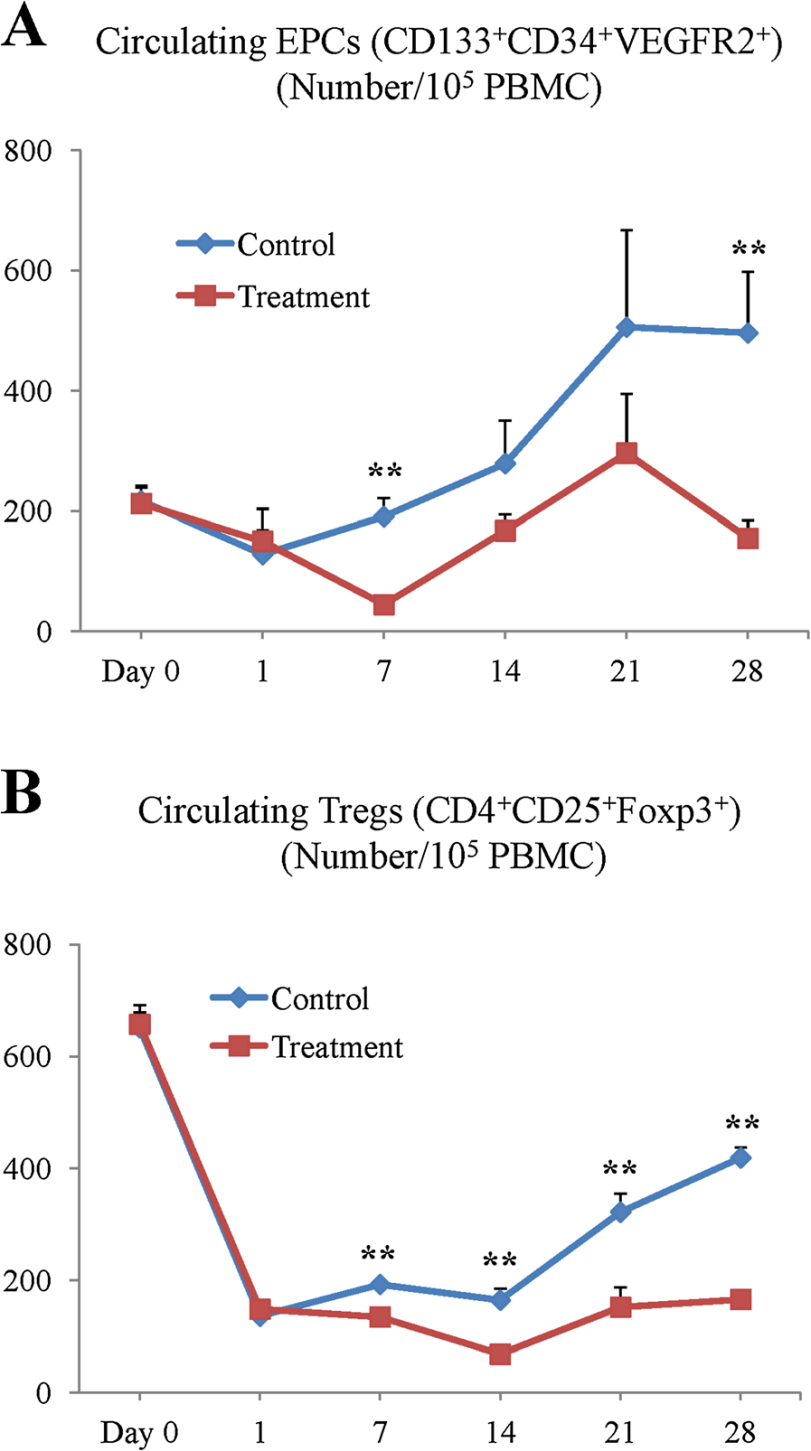
**Comparison of circulating EPCs and Tregs.** The populations of circulating EPCs and Tregs were detected by flow cytometry at day 0, 1, 7, 14, 21, 28 after major hepatectomy and I/R injury. **(A)** The level of circulating EPCs was compared between YQ23 treatment and control groups. **(B)** The number of circulating Tregs was detected in YQ23 treatment and control groups. (**p < 0.01; N = 3/group).

EPCs play important roles in tumor vasculogenesis and tumor growth at early phase by providing structural support to nascent vessels and the release of pro-angiogenic cytokines. In order to explore the underlying mechanism of YQ23 on suppressing liver tumor metastasis, we detected the levels of the circulating EPCs at different time points after major hepatectomy and I/R injury. The level of circulating EPCs was decreased at day 1 after the major hepatectomy and partial I/R injury in both control and treatment group. After that, compared to control group, the level of circulating EPCs was significantly reduced after YQ23 treatment at day 7 and day 28 (day7: 44.6 *vs* 190.9/10^5^ PBMC cells, *p = 0.001*; day28: 155.0 *vs* 496.6/10^5^ PBMC cells, *p = 0.005*) (Figure 3A). At day 14 and day 21, the treatment of YQ23 also effectively decreased the circulating EPCs levels (day14: 167.4 *vs* 278.8/10^5^ PBMC cells, *p = 0.06*; day21: 296.1 *vs* 506.2/10^5^ PBMC cells, *p = 0.13*) (Figure 3A).

### YQ23 significantly reduced circulating Tregs

Tregs play a critical role in antitumor immune responses. They can suppress the immune responses against tumors and thereby promote tumor growth. The results showed that the number of circulating Tregs was reduced at day 1 after the major hepatectomy and partial I/R injury in both groups. The level of circulating Tregs was significantly decreased by YQ23 treatment from day 7 to day 28 (day7: 134.6 *vs* 192.9/10^5^ PBMC cells, *p = 0.003*; day14: 68.8 *vs* 164.7/10^5^ PBMC cells, *p = 0.002*; day21: 153.2 *vs* 322.2/10^5^ PBMC cells, *p = 0.004*; day28: 165.9 *vs* 419.4/10^5^ PBMC cells, *p < 0.001*) (Figure 3B).

### Administration of YQ23 increased liver pO_2_ during ischemia and reperfusion process

**Figure 4:**
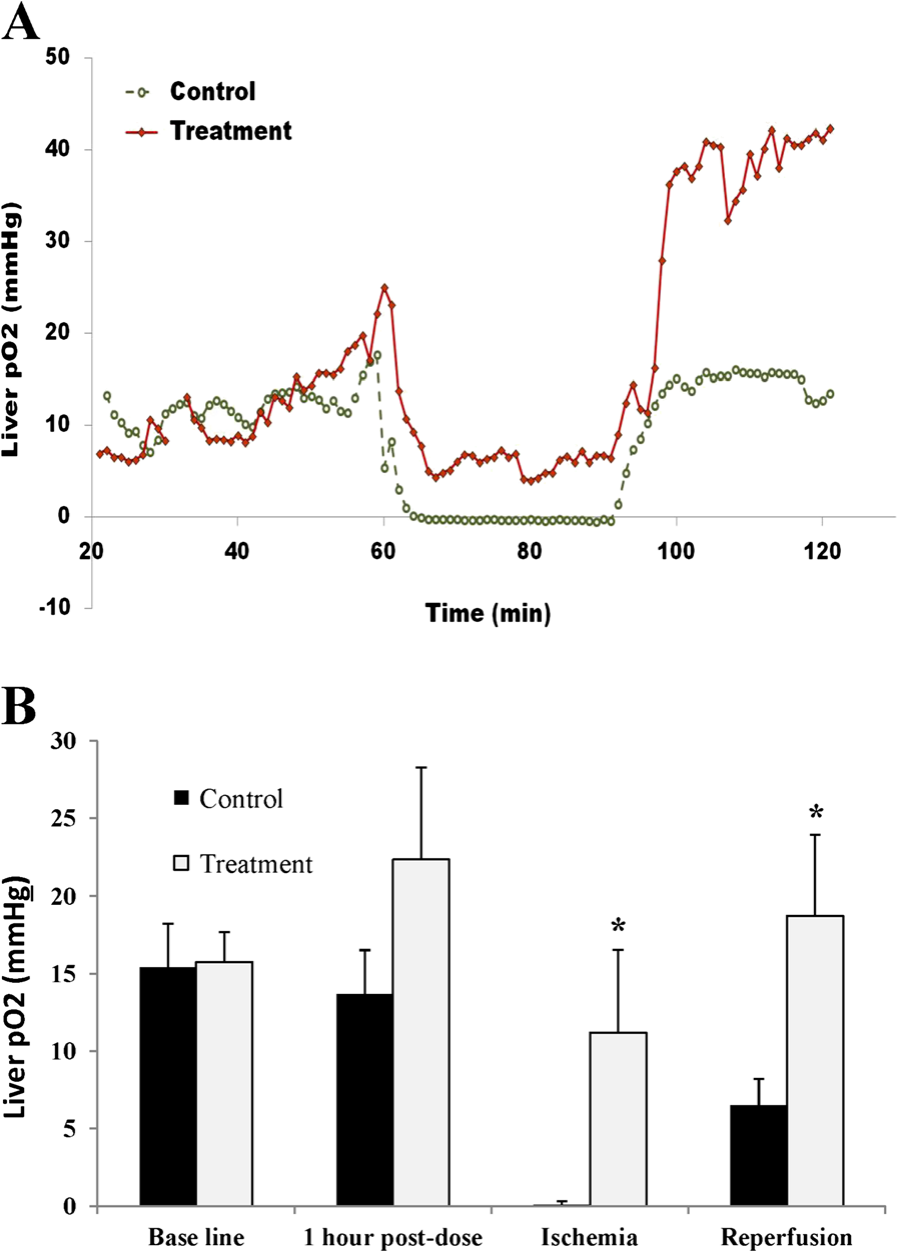
**YQ23 increased liver pO**_**2 **_**during hepatic I/R procedure.** Liver pO_2_ level was directly monitored by the OxyLab® *in vivo* monitoring system during the ischemia and reperfusion procedures. **(A)** Representative liver pO_2_ curves during ischemia reperfusion procedures were presented. **(B)** Average liver pO_2_ level was showed at different time points during ischemia reperfusion procedures. (*p < 0.05; Treatment group N = 6; Control group N = 7).

Accumulating evidence indicated that tissue ischemia and hypoxia, through elevated levels of HIF-1α responsive chemokines such as SDF-1α and VEGF, stimulate the release and recruitment of EPCs and Tregs from the bone marrow. In order to explore the underlying mechanism of YQ23 on suppression of circulating EPCs and Tregs, pO2 levels in liver tissue during hepatic IR injury were compared between control and treatment group. Representative continuous measurement of liver pO2 in rats following intravenous administration of YQ23 and control during the ischemia reperfusion procedures was presented in Figure 4A. At baseline, the average liver pO_2_ did not show significant difference between the treatment and control group (15.7 *vs* 15.4 mmHg; *p* = 0.93). One hour after YQ23 or buffer injection, YQ23 treatment effectively increased liver pO_2_ level (22.3 *vs* 13.7 mmHg, *p* = 0.19) although the values did not reach a statistical significant level (Figure 4B). During ischemia, although liver pO_2_ was decreased in both treatment and control group, YQ23 treatment resulted in a significantly higher pO_2_ (11.2 mmHg) when compared with control group (0.1 mmHg, *p* = 0.045). After reperfusion, the pO_2_ level in liver was also found to be elevated in YQ23 treatment group (18.7 *vs* 6.5, *p* = 0.036) (Figure 4B).

### YQ23 treatment increased HO1 expression and down-regulated the expressions of CXCR3, TNF-α and IL6

**Figure 5:**
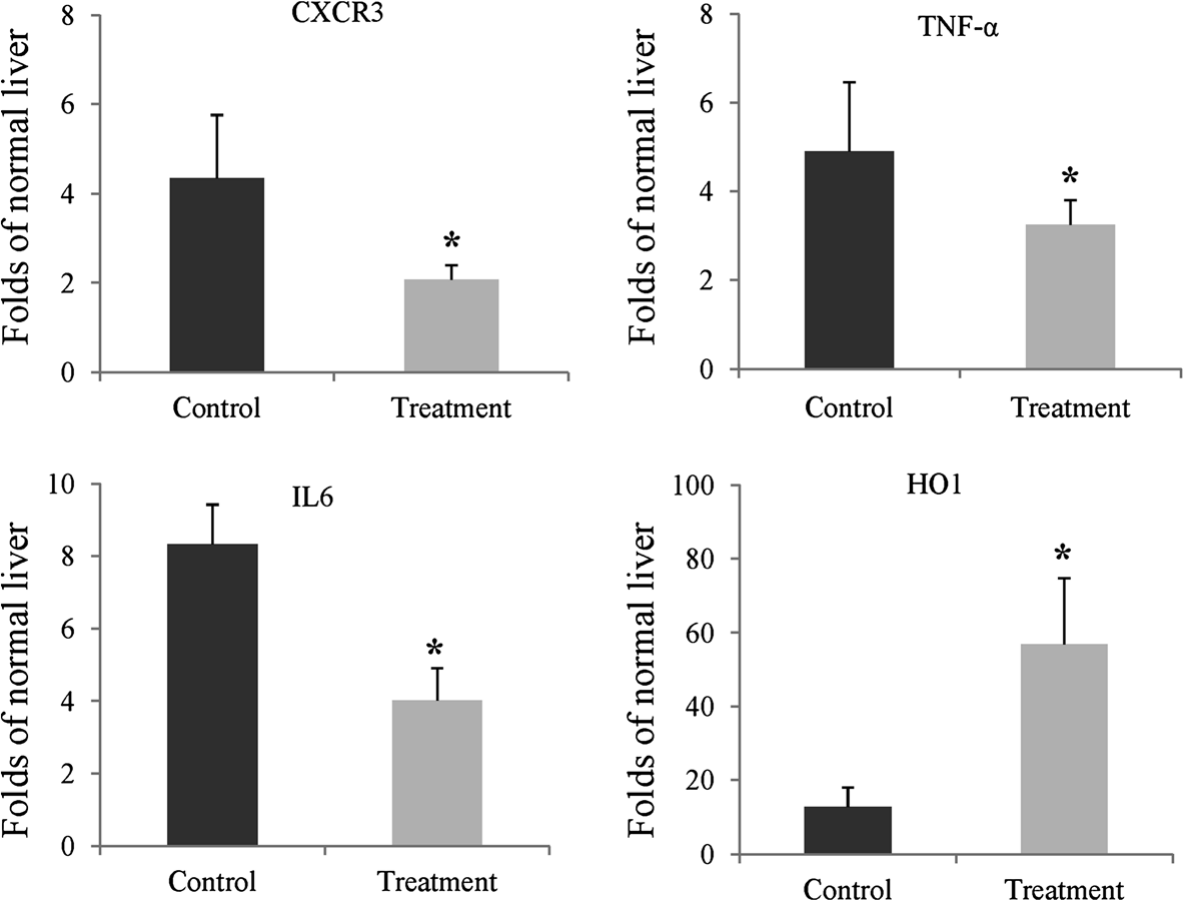
**YQ23 increased HO1 expression and down-regulated the expressions of CXCR3, TNF-α and IL6.** Hepatic gene expressions were detected by real time RT-PCR at 6 hours after major hepatectomy and hepatic I/R injury. *P < 0.05; N = 4/group.

In order to explore the underlying mechanism of YQ23 on suppression of circulating EPCs and Tregs, the mRNA expression levels of CXCR3, TNF-α, IL6 and HO1 in liver tissue were compared between control group and treatment group. YQ23 significantly increased HO1 expression and down-regulated expressions of CXCR3, TNF-α and IL6 in liver tissue at 6 hours after major hepatectomy and hepatic I/R injury compared to control group (Figure 5).

## Discussion and conclusions

In the present study, the effect of YQ23 on inhibition of liver tumor metastasis after liver resection and I/R injury was first shown in an orthotopic rat liver tumor model with local and distant metastatic potentials. Although the incidence of tumor metastasis in both intrahepatic and lung after YQ23 treatment didn’t reach a statistical difference level, the incidence was reduced by approximately 50% (From 69.2% to 36.4% in liver; from 53.8% to 36.4% in lung). In addition to reducing the incidence of intraheptic and lung metastasis, YQ23 also decreased the number and size of metastatic tumor nodules at four weeks after major hepatectomy and hepatic I/R injury. These results suggest that the YQ23 treatment may provide a novel means to prevent liver cancer metastasis after liver surgery for HCC patients.

Liver ischemia and hypoxia are inevitable consequence due to operation or clamp during liver surgery. It can lead to a set of liver damage and change in micro environment. Accumulating evidence indicated that tissue ischemia and hypoxia induced the production of HIF-1*α* responsive chemokines and inflammatory cytokines/chemokines [4-6]. They play important roles in stimulating the release and recruitment of EPCs and Trges from the bone marrow [6-10]. Recently, we have also demonstrated that post-transplant endothelial progenitor cell mobilization via CXCL10/CXCR3 signaling promoted liver tumor growth [10]. In this study, our results showed that YQ23 treatment decreased CXCR3 expression after I/R injury. The reduction of circulating EPCs and Tregs levels may due to the down-regualation of CXCR3 after I/R injury. Furthermore, YQ23 treatment also decreased the expressions of TNF-α and IL6, which play important roles in the pathogenesis of hepatic I/R injury. On the other hand, YQ23 treatment increased liver pO_2_ level during operation and HO1 expression after hepatic I/R injury. A wealth of data has proven that HO1 and its metabolites protect liver from hepatic I/R injury through modulating inflammatory responses and anti-oxidant/oxidant homeostasis [23].

Bone marrow derived EPCs have the capacity to migrate to the peripheral circulation and differentiate into mature endothelial cells [24,25]. Several studies have demonstrated that EPCs play important roles in the development of tumor vasculature. They contribute to early-stage tumor vascularization, promote tumor growth and control angiogenic switch of metastasis transition [11-13]. Furthermore, the ablation of EPCs mobilization or integaration can result in significant angiogenesis inhibition and impaired tumor growth and metastasis [26,27]. In this study, our results showed that YQ23 reduced the number of circulating EPCs at different time point after liver tumor resection and I/R injury. The phenomenon was consistent with the decrease of MVD in metastatic tumor nodules.

In addition to the reduction of circulating EPCs levels, the present study also demonstrated that YQ23 decreased the levels of circulating Tregs after liver resection and hepatectomy. Recent studies have shown that CD4^+^CD25^+^Foxp3^+^ Tregs, which are physiologically engaged in the maintenance of immunological self-tolerance and immune homeostasis, play a critical role in the control of antitumor immune responses [14,15]. It has been found that increased numbers of Tregs are detected in peripheral blood of cancer patients and accumulate in tumor regions [16-18]. Furthermore, deletion or inhibition of Tregs effectively enhanced antitumor responses and inhibited tumor growth [28,29]. Therefore, the effect of YQ23 on inhibition of liver tumor metastasis was probably due to the decrease of circulating EPCs and Tregs levels. Targeting circulating EPCs and Tregs by YQ23 treatment may effectively decrease tumor metastasis after liver surgery.

In conclusion, YQ23 suppressed liver tumor metastasis after major hepatectomy and partial hepatic I/R injury through increasing liver oxygenation and reducing the number of circulating EPCs and Tregs, suggesting that it may be a promising candidate for potential adjuvant therapies for treating liver cancer metastasis after liver surgery for HCC patients.

## Competing interests

The co-authors Bing L. Wong, Sze Hang Lau, Sui Yi Kwok, Na Wei and Fei Chuen Tzang were under the employment of “New B Innovation Ltd” at the time when these data were published. Other authors have not conflict of interest.

## Authors’ contributions

KM, CML and BLW conceived of the research. CXL, CCL, YYM, KTPN, XBL, YS, WG, XQ, NW, FCT performed the research. CXL drafted the manuscript. SHL and SYK revised the manuscript. All authors read and approved the final manuscript.

## Pre-publication history

The pre-publication history for this paper can be accessed here:

http://www.biomedcentral.com/1471-2407/14/293/prepub

## References

[B1] Bosch FX Ribes J Diaz M Cleries R Primary liver cancer: worldwide incidence and trends Gastroenterology 2004 127 5 Suppl 1 S5 S16 15508102 10.1053/j.gastro.2004.09.011

[B2] Shah SA Cleary SP Wei AC Yang I Taylor BR Hemming AW Langer B Grant DR Greig PD Gallinger S Recurrence after liver resection for hepatocellular carcinoma: risk factors, treatment, and outcomes Surgery 2007 141 3 330 339 10.1016/j.surg.2006.06.028 17349844

[B3] Sakon M Umeshita K Nagano H Eguchi H Kishimoto S Miyamoto A Ohshima S Dono K Nakamori S Gotoh M Monden M Clinical significance of hepatic resection in hepatocellular carcinoma: analysis by disease-free survival curves Arch Surg 2000 135 12 1456 1459 10.1001/archsurg.135.12.1456 11115352

[B4] Takahashi T Kalka C Masuda H Chen D Silver M Kearney M Magner M Isner JM Asahara T Ischemia- and cytokine-induced mobilization of bone marrow-derived endothelial progenitor cells for neovascularization Nat Med 1999 5 4 434 438 10.1038/7434 10202935

[B5] Lemoli RM Catani L Talarico S Loggi E Gramenzi A Baccarani U Fogli M Grazi GL Aluigi M Marzocchi G Bernardi M Pinna A Bresadola F Baccarani M Andreone P Mobilization of bone marrow-derived hematopoietic and endothelial stem cells after orthotopic liver transplantation and liver resection Stem Cells 2006 24 12 2817 2825 10.1634/stemcells.2006-0333 16931769

[B6] Clambey ET McNamee EN Westrich JA Glover LE Campbell EL Jedlicka P De Zoeten EF Cambier JC Stenmark KR Colgan SP Eltzschig HK Hypoxia-inducible factor-1 alpha-dependent induction of FoxP3 drives regulatory T-cell abundance and function during inflammatory hypoxia of the mucosa Proc Natl Acad Sci U S A 2012 109 41 E2784 E2793 10.1073/pnas.1202366109 22988108 PMC3478644

[B7] Kalka C Masuda H Takahashi T Gordon R Tepper O Gravereaux E Pieczek A Iwaguro H Hayashi SI Isner JM Asahara T Vascular endothelial growth factor(165) gene transfer augments circulating endothelial progenitor cells in human subjects Circ Res 2000 86 12 1198 1202 10.1161/01.RES.86.12.1198 10864908

[B8] Ben-Shoshan J Maysel-Auslender S Mor A Keren G George J Hypoxia controls CD4 + CD25+ regulatory T-cell homeostasis via hypoxia-inducible factor-1alpha Eur J Immunol 2008 38 9 2412 2418 10.1002/eji.200838318 18792019

[B9] Chang EI Thangarajah H Hamou C Gurtner GC Hypoxia, hormones, and endothelial progenitor cells in hemangioma Lymphat Res Biol 2007 5 4 237 243 10.1089/lrb.2007.1014 18370914

[B10] Ling CC Ng KT Shao Y Geng W Xiao JW Liu H Li CX Liu XB Ma YY Yeung WH Qi X Yu J Wong N Zhai Y Chan SC Poon RT Lo CM Man K Post-transplant endothelial progenitor cell mobilization via CXCL10/CXCR3 signaling promotes liver tumor growth J Hepatol 2014 60 1 103 109 10.1016/j.jhep.2013.08.017 23994383

[B11] Peters BA Diaz LA Polyak K Meszler L Romans K Guinan EC Antin JH Myerson D Hamilton SR Vogelstein B Kinzler KW Lengauer C Contribution of bone marrow-derived endothelial cells to human tumor vasculature Nat Med 2005 11 3 261 262 10.1038/nm1200 15723071

[B12] Lyden D Hattori K Dias S Costa C Blaikie P Butros L Chadburn A Heissig B Marks W Witte L Wu Y Hicklin D Zhu Z Hackett NR Crystal RG Moore MA Hajjar KA Manova K Benezra R Rafii S Impaired recruitment of bone-marrow-derived endothelial and hematopoietic precursor cells blocks tumor angiogenesis and growth Nat Med 2001 7 11 1194 1201 10.1038/nm1101-1194 11689883

[B13] Gao D Nolan DJ Mellick AS Bambino K McDonnell K Mittal V Endothelial progenitor cells control the angiogenic switch in mouse lung metastasis Science 2008 319 5860 195 198 10.1126/science.1150224 18187653

[B14] Sakaguchi S Yamaguchi T Nomura T Ono M Regulatory T cells and immune tolerance Cell 2008 133 5 775 787 10.1016/j.cell.2008.05.009 18510923

[B15] Shimizu J Yamazaki S Sakaguchi S Induction of tumor immunity by removing CD25 + CD4+ T cells: a common basis between tumor immunity and autoimmunity J Immunol 1999 163 10 5211 5218 10553041

[B16] Woo EY Chu CS Goletz TJ Schlienger K Yeh H Coukos G Rubin SC Kaiser LR June CH Regulatory CD4(+)CD25(+) T cells in tumors from patients with early-stage non-small cell lung cancer and late-stage ovarian cancer Cancer Res 2001 61 12 4766 4772 11406550

[B17] Wolf AM Wolf D Steurer M Gastl G Gunsilius E Grubeck-Loebenstein B Increase of regulatory T cells in the peripheral blood of cancer patients Clin Cancer Res 2003 9 2 606 612 12576425

[B18] Feng X Li B Ye H Long D Increased frequency of CD4 + CD25(high)FoxP3+ regulatory T cells in patients with hepatocellular carcinoma Arch Immunol Ther Exp 2011 59 4 309 314 10.1007/s00005-011-0127-0 21633918

[B19] Liu XB Cheng Q Geng W Ling CC Liu Y Ng KT Yam JW Guan XY Lo CM Man K Enhancement of cisplatin-based TACE by a hemoglobin-based oxygen carrier in an orthotopic rat HCC model Artif Cells Nanomed Biotechnol 2013 Epub ahead of print 10.3109/21691401.2013.808647 23795724

[B20] Li CX Shao Y Ng KT Liu XB Ling CC Ma YY Geng W Fan ST Lo CM Man K FTY720 suppresses liver tumor metastasis by reducing the population of circulating endothelial progenitor cells PLoS One 2012 7 2 e32380 10.1371/journal.pone.0032380 22384233 PMC3288101

[B21] Man K Ng KT Xu A Cheng Q Lo CM Xiao JW Sun BS Lim ZX Cheung JS Wu EX Sun CK Poon RT Fan ST Suppression of liver tumor growth and metastasis by adiponectin in nude mice through inhibition of tumor angiogenesis and downregulation of Rho kinase/IFN-inducible protein 10/matrix metalloproteinase 9 signaling Clin Cancer Res 2010 16 3 967 977 10.1158/1078-0432.CCR-09-1487 20103676

[B22] Man K Ng KT Lo CM Ho JW Sun BS Sun CK Lee TK Poon RT Fan ST Ischemia-reperfusion of small liver remnant promotes liver tumor growth and metastases–activation of cell invasion and migration pathways Liver Transpl 2007 13 12 1669 1677 10.1002/lt.21193 18044786

[B23] Shen XD Ke B Zhai Y Gao F Busuttil RW Cheng G Kupiec-Weglinski JW Toll-like receptor and heme oxygenase-1 signaling in hepatic ischemia/reperfusion injury Am J Transplant 2005 5 8 1793 1800 10.1111/j.1600-6143.2005.00932.x 15996225

[B24] Rookmaaker MB Tolboom H Goldschmeding R Zwaginga JJ Rabelink TJ Verhaar MC Bone-marrow-derived cells contribute to endothelial repair after thrombotic microangiopathy Blood 2002 99 3 1095 10.1182/blood.V99.3.1095 11822359

[B25] Walter DH Rittig K Bahlmann FH Kirchmair R Silver M Murayama T Nishimura H Losordo DW Asahara T Isner JM Statin therapy accelerates reendothelialization: a novel effect involving mobilization and incorporation of bone marrow-derived endothelial progenitor cells Circulation 2002 105 25 3017 3024 10.1161/01.CIR.0000018166.84319.55 12081997

[B26] Nozawa H Chiu C Hanahan D Infiltrating neutrophils mediate the initial angiogenic switch in a mouse model of multistage carcinogenesis Proc Natl Acad Sci U S A 2006 103 33 12493 12498 10.1073/pnas.0601807103 16891410 PMC1531646

[B27] Shojaei F Wu X Malik AK Zhong C Baldwin ME Schanz S Fuh G Gerber HP Ferrara N Tumor refractoriness to anti-VEGF treatment is mediated by CD11b + Gr1+ myeloid cells Nat Biotechnol 2007 25 8 911 920 10.1038/nbt1323 17664940

[B28] Dannull J Su Z Rizzieri D Yang BK Coleman D Yancey D Zhang A Dahm P Chao N Gilboa E Vieweg J Enhancement of vaccine-mediated antitumor immunity in cancer patients after depletion of regulatory T cells J Clin Invest 2005 115 12 3623 3633 10.1172/JCI25947 16308572 PMC1288834

[B29] Litzinger MT Fernando R Curiel TJ Grosenbach DW Schlom J Palena C IL-2 immunotoxin denileukin diftitox reduces regulatory T cells and enhances vaccine-mediated T-cell immunity Blood 2007 110 9 3192 3201 10.1182/blood-2007-06-094615 17616639 PMC2200901

